# Radiological and clinical outcomes in patients undergoing anterior cervical discectomy and fusion: Comparing titanium and PEEK (polyetheretherketone) cages

**DOI:** 10.12669/pjms.346.15833

**Published:** 2018

**Authors:** Muhammad Junaid, Mamoon Ur Rashid, Syed Sarmad Bukhari, Mamoon Ahmed

**Affiliations:** 1 *Dr. Muhammad Junaid, FCPS. Associate Professor Head of Neurosurgery, PNS Shifa, Karachi, Pakistan*; 2 *Dr. Mamoon Ur Rashid, MBBS. Department of Internal Medicine, Orlando Hospital, Orlando, FL, USA*; 3 *Dr. Syed Sarmad Bukhari, MBBS. Resident Neurosurgery, Aga Khan University Hospital, Karachi. Pakistan*; 4 *Dr. Mamoon Ahmed, MBBS. House Officer, Jinnah Hospital, Lahore, Pakistan*

**Keywords:** Axial neck pain, Cervical discectomy, Titanium cage, PEEK cage

## Abstract

**Objectives:**

To study clinical and radiological outcomes in patients who had undergone the procedure of anterior cervical discectomy and fusion with titanium or PEEK (polyetheretherketone) cages for cervical disc prolapse.

**Methods:**

This is a retrospective/non-randomized study which was conducted at the Combined Military Hospital Peshawar. Study interval was four years from 1st October, 2010 to 31st September, 2014. Total number of included patients were 149. All of the patients had undergone the procedure of anterior cervical discectomy and fusion with titanium or PEEK (polyetheretherketone) cages. All of the patients had plain MRI cervical spine done for diagnosis of anterior cervical disc prolapse.

**Results:**

Most of the patients had stenosis at the C5 / C6 (PEEK cage group 63% and titanium cage group 47.6%) and C6 / C7 (PEEK cage group 15.38% and titanium cage group 19.04%) cervical level. Bi-level involvement was also seen. In the patients who complained of brachialgia, total resolution of symptoms was seen after the operation. Three (2.01%) of the patients in titanium cage group, who presented with axial neck pain, continued to complain of pain after the operation. Four (2.6%) of the patients in PEEK (polyetheretherketone) cage group and 2 (1.3%) in titanium cage group complained of pain at the donor site (iliac crest). Fusion rate was 100% with both titanium and PEEK (polyetheretherketone) cages at one year.

**Conclusion:**

Results with titanium and PEEK (polyetheretherketone) cages are excellent. There was no significant difference in clinical and radiological outcome between two groups of patients (p > 0.05). Fusion rate was 100% at one year with both cages.

## INTRODUCTION

Disc degeneration is one of the causes for intervertebral disc prolapse, for which both degenerative disc disease and aging are important.^1^ Disc prolapse leads to compression of nerves and spinal cord due to which symptoms arise. Now there is also evidence of chemical inflammation^2^-^5^ in the causation of symptoms.

Anterior cervical decompression and fusion is a procedure used for degenerative cervical diseases first reported in 1958. Most authors agree that discectomy should be combined with interbody fusion. The gold standard is a cancellous bone graft taken from the iliac crest.^6^ The auto graft taken from the iliac bone is associated with most of the complications like postoperative pain, hematoma formation, longer hospital stays, infection, longer operative time. The complication rate might range from 9.4 to 50% with this procedure.^7^

Anterior cervical discectomy along with fusion is combined with anterior cervical plating for multilevel prolapses. However complications have also been reported with these procedures. The various complications which might develop during anterior cervical discectomy and fusion, includes cage breakage, plate migration, stress shielding, compression of tissues, spinal cord and nerves, which might require surgery.^8^ Several type of cages have been developed and used for fusion in clinical practice.^9^

Peek and titanium cages are used in our setup. Studies showed promising results after the fusion with titanium cages.^10^,^11^ Some of the studies have shown the comparison of peek and titanium cages with respect to clinical and radiological outcome.^12^,^13^ A couple of studies showed no significant difference between peek and titanium cages while some showed peek cages to be superior to titanium cages in maintaining interspace height and cervical fusion but regarding clinical outcomes results were comparable between two groups. We attempted to determine differences in outcomes between the two methods in our setup.

## METHODS

This is a retrospective study which was conducted in Combined Military Hospital of Peshawar. Study duration was four years from 1^st^ October, 2010 to 31^st^ September, 2014. Patients who presented, were investigated and operated were included. All of the patients with clinical and radiologic evidence of intervertebral disc prolapse who underwent surgery were included while the patients who had radiculopathy or myelopathy due to any other reason than cervical disc prolapse were excluded. These patients had also failed to improve significantly on conservative treatment with neuropathic pain medicines (pregabalin), analgesics (naproxen), muscle relaxants (baclofen), vitamins (B1, B6 and B12), soft neck collars and physiotherapy for 6 weeks. MRI was the investigation of choice. Total number of patients was 149 who belonged to various cities of Khyber Pukhtunkhwa.

The standard treatment of cervical disc prolapse surgery was done, which included discectomy including removal of posterior longitudinal ligament. For fusion PEEK and TITANIUM cages were used which were filled with cancellous graft from right iliac crest. PEEK stands for Polyetheretherketone, which is a colorless thermoplastic polymer which stimulates osteoblastic and inhibits osteoclastic activity. Titanium has a high strength, low density and is quite resistant to corrosion. It has no effect on osteoblastic and osteoclastic activity. Patients with osteopenia on cervical spine x-ray done were treated with Calcium supplements before surgery.

Previous research showed that there was no significant difference between the outcomes of procedure which used PEEK and titanium cages after cervical disc prolapsed surgery.^14^ This fact was discussed with the patients and fully informed consent was obtained. Mostly, patients were from low socioeconomic class and preferred titanium cages as PEEK cages were expensive. On this basis, patients who were operated were divided in to 2 groups, PEEK cage group and Titanium cage group.

All the patients were followed for one year with cervical spine x-rays to assess fusion, cage migration, subsidence or breakage at two weeks, six weeks, six months and one year after surgery. The parameters noted on follow up were: pain, personal care, reading, headaches, concentration, driving, sleeping and recreation. Complications which were noted were included in the results.

Chi-square test was used to assess the surgical (fusion) outcome at six months and one year. P value was set at 0.05%. Null hypothesis was formulated that there was no statistical significance between outcomes of two groups.

## RESULTS

Total numbers of patients were 149. Males were 98 (65.77%) and females were 51 (34.22%). Male to female ratio was 1.92:1. The patients presented with following signs and symptoms: axial neckache, brachialgia, myelopathy, and poor hand grip/numbness. Most commonly noted complaint was brachialgia in both groups (PEEK cage group 60% and titanium cage group 48.8%)., followed by myelopathy in both cage groups (PEEK cage group 24.6% and titanium cage group 34.5%). The various level of stenosis noted on plain MRI of cervical spine are given in the Table-I. Most commonly involved cervical intervertebral spaces were C5 / C6 (PEEK cage group 63% and titanium cage group 47.6% and C6 / C7 (PEEK cage group 15.38% and titanium cage group 19.04%). Bilateral involvement was seen in both age groups. C5/C6 and C6/C7 were commonly involved bi-levels in both age groups. 79 (53.02%) patients had moderate disability, 42 (28.18%) had severe disability and 28 (18.79%) had mild disability according to neck disability index (NDI). The details are given in the Table-I.

**Table-I:** Patient characteristics in both groups.

Parameters	PEEK cage group	Titanium cage group
Mean age	36 years (26-65).	45.9 years (20-70)
Male/Female	44 / 21	54 / 30
Total	65	84
Unilateral brachialgia	39	41
Bilateral brachialgia	4	6
Myelopathy	16	29
Axial neck pain	3	6
Numbness/Poor grip	3	2
Single/Double level	59 / 6	68 / 16
C3/C4	1	2
C4/C5	7	8
C5/C6	41	40
C6/C7	10	16
C7 / T1	0	2
C3/C4, C4/C5	1	4
C4/C5, C5/C6	1	4
C5/C6, C6/C7	4	8

### Titanium cage group

Mean age of the patients in Titanium cage group was 45.9 years ranging from 20 – 70 years. To assess the fusion and to note the complications patients were regularly followed and cervical x-rays were carried at four intervals. 1st x-ray was done at the operation time, 2nd at six weeks interval, 3rd at six months and 4th at one year interval from operation day. Patients were regularly followed to assess fusion, cage breakage, plate migration, stress shielding, compression of tissues, spinal cord or nerves.

**Fig.1:**
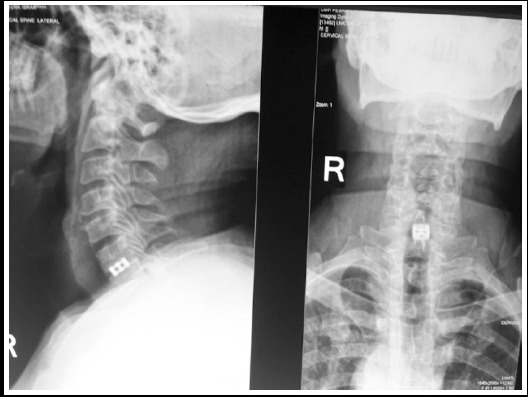
Fusion with titanium cage on cervical X-ray at C6/C7 spinal level at 1 year of follow up.

Fusion rate at six months was 55% and at one year was 100%. Both clinical and radiological improvement was seen in the patients. In patients with brachialgia, total improvement was seen but in the patients who were complaining of axial neck pain, 3(2.01%)of the them continued to complain of pain. Pain at the donor site was reported by two (1.3%) patients who responded to neuropathic pain medicine (pregabalin). All the patients with radiculopathy were advised rest for six weeks and then to return to normal daily activities.

### PEEK cage group

Mean age of the patients in PEEK cage group was 36 years ranging from 26 – 65 years For follow up, X rays were done at intervals similar to the titanium cage group i.e 1st x ray was done at the operation time, 2nd at 6 weeks interval, 3rd at 6 months and last one at 1 year interval from operation day. Patients were regularly followed to assess fusion cage breakage, plate migration, subsidence, compression of tissues, spinal cord or nerves.

Fusion rate at 6 months was 60% and at 1 year was 100%. Both clinical and radiological improvement was seen in the patients. In patients symptoms of brachialgia and axial neck pain completely resolved. 4 (2.6%) patients complained of pain at donor site which was treated with pregabalin and response was good. All the patients with radiculopathy were advised rest for six weeks and then to return to normal daily activities.

**Fig.2:**
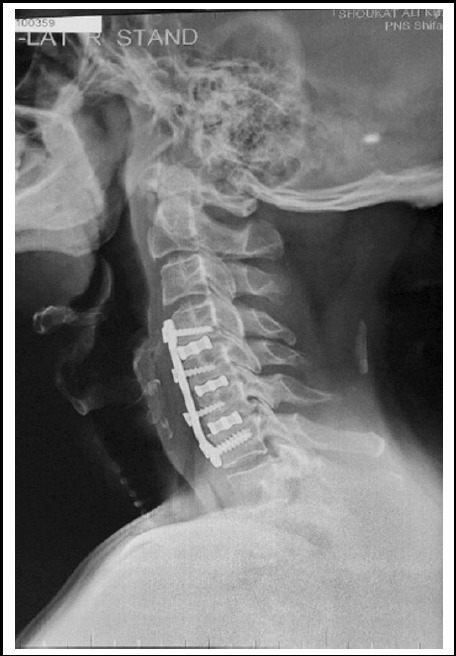
Shows tri level ACDF and plating with titanium cages.

**Table:** 

	Fusion at 6 months	Non fusion at 6 months
PEEK cage group	60% of patients	40% of patients
Titanium cage group	55% of patients	45% of patients

**Table:** 

	Fusion at 1 year	Non fusion at 1 year
PEEK cage group	100 % of patients	0 % of patients
Titanium cage group	100 % of patients	0 % of patients

Calculated value by Chi-square test at 6 months was 0.52. It was less than 3.84 with 95% confidence interval and 1 degree of freedom.

Since fusion rate was 100% at the end of one year. Calculated value by Chi-square test at one year was 0. It was less than 3.84 with 95% confidence interval and 1 degree of freedom. Null hypothesis could not be rejected. There was no statistical significance between the outcomes of two groups.

**Fig.3:**
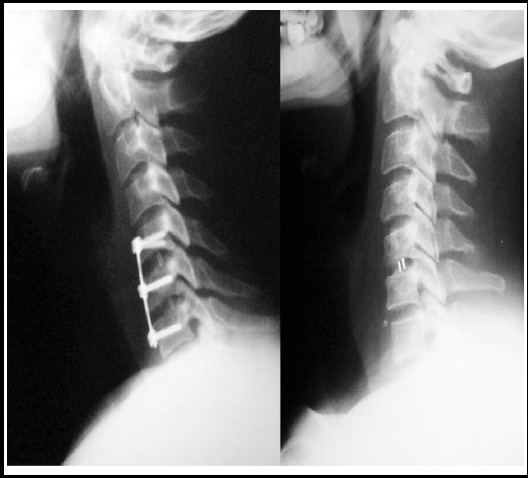
Cage intact in multilevel discectomy. Bi level PEEK cages visible on lateral x rays in this two level fixation with anterior plating (Left). Single level PEEK ACDF at C5,6 (Right).

## DISCUSSION

Anterior cervical discectomy is a common procedure performed for degenerative spinal diseases. After this procedure for stabilization of cervical vertebrae bone autografts and bioresorbable cages are used. One of the study by Xie JC and Hurlbert R^15^ tried to compare the fusion rate after anterior cervical discectomy with and without bone grafts and bioresorbable cages. The following results were obtained.

- Fusion rate without bone graft and cages was 67%.
- Fusion rate with bone graft was 93%.
- Fusion rate with cages was 100%.
- Another study by Mobbs RJ, Rao P, & Chandra NK^16^, showed following results
- 98% fusion was noted in the plating group.
- 93.5% fusion was noted in the non-plating group.

Similarly some of the studies performed alone with titanium cages^17^ and peek cages^18^ showed good results. Study by Hwang et al^17^ showed fusion rate of 96.3% with titanium cages and 91.4% with bone grafts. A recently published study showed the fusion rate with a porous PEEK interbody fusion device to be 100% at 12 months.^19^

Some of the studies tried to compare the results of titanium and PEEK cages used in anterior cervical discectomy.^20^ Study by Cabraja et al^21^ showed no significant difference between peek and titanium cages but study by Niu et al^22^ showed peek cages to be superior to titanium cages. Study by Cabraja et al^21^ showed that solid arthrodesis was found in 93.2% of the titanium group and 88.1% of the PEEK group. Niu et al^22^ reported that the fusion rate was higher in the PEEK group and was 100% vs. 86.5% in titanium group. In the surgical treatment of multilevel cervical spondylotic myelopathy, PEEK cages are superior to titanium cages in maintaining cervical lordosis and intervertebral height, resulting in good clinical results.^23^

**Fig.4:**
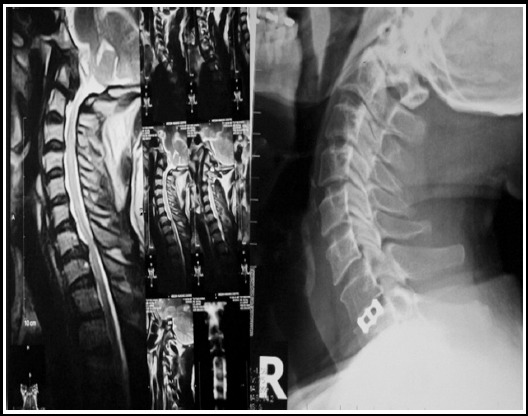
Preop MRI showing c7/T1 prolapse disc(Right) post op x-ray showing Titanium cage(Left).

In our study we tried to compare the results of ACDF procedure with titanium and PEEK cages. The follow up X-rays showed 100% fusion rate at one year in both cage group. Some of the complications of surgery are complications of anesthesia, hemorrhage, wound hematoma, damage to the carotid or vertebral artery, damage to the recurrent laryngeal nerve, damage to the superior laryngeal nerve, damage to the esophagus or trachea, damage to the dura, wound infection, development of painful pseudoarthrosis, damage to the spinal cord or nerve root. Only one patient in our study developed subcutaneous hematoma in PEEK cage group which was successfully treated.

No complication of breakage, subsidence or cage migration was observed in our patients. Cervical inter-body fusion was uneventful continuing to present. The main goal of cervical spine surgery was to ensure that nerve compression is relieved, fusion has occurred and cervical spine is stable.

The patients presented with axial neckache, brachilagia, quadriparesis, monoparesis and poor hand grip. Most commonly noted complaint was brachialgia in both groups (PEEK cage group 60% and titanium cage group 48.8%)., followed by myelopathy in both cage groups (PEEK cage group 24.6% and titanium cage group 34.5%). Most commonly involved cervical intervertebral spaces were C5 / C6 and C6 / C7 (19%). Most commonly involved cervical intervertebral spaces were C5 / C6 (PEEK cage group 63% and titanium cage group 47.6%) and C6 / C7 (PEEK cage group 15.38% and titanium cage group 19.04%).

Recovery of symptoms was good in titanium cage group but three (2.01%) of the patients continued to have the axial neck pain in spite of cage transplant and no cause was identified 2 (1.3%) of patients from Titanium cage group and 4 (2.6%) patients from Peek cage group were complaining of pain at the donor site. Pain responded to neuropathic pain medicine (pregabalin).

## CONCLUSION

The results of anterior cervical discectomy and fusion with PEEK and titanium cages in our institution are excellent with outcomes similar to internationally reported studies. The fusion rate was 100% at one year with both cages. The improvement of symptoms of brachialgia was 100%, while few of the patients with axial neck pain, continued to have the symptoms.

### Authors’ Contribution

**MJ:** Contribution to conception, design and acquisition of data, Final approval of the article.

**MUR:** Conception and design, revision of article for intellectual content, Final approval of the article.

**SSB:** Analysis of data, revision of manuscript and final approval.

**MA:** Conception and design, drafting of article, revision of article for intellectual content, Final approval of the article.
